# Machine Learning Assisted Cervical Cancer Detection

**DOI:** 10.3389/fpubh.2021.788376

**Published:** 2021-12-23

**Authors:** Mavra Mehmood, Muhammad Rizwan, Michal Gregus ml, Sidra Abbas

**Affiliations:** ^1^Department of Computer Science, Kinnaird College for Women, Lahore, Pakistan; ^2^Information Systems Department, Faculty of Management, Comenius University in Bratislava, Bratislava, Slovakia; ^3^ASETS Lab, Islamabad, Pakistan

**Keywords:** cervical cancer, medical data, gynecological diseases, artificial intelligence, feature engineering, classification

## Abstract

Cervical malignant growth is the fourth most typical reason for disease demise in women around the globe. Cervical cancer growth is related to human papillomavirus (HPV) contamination. Early screening made cervical cancer a preventable disease that results in minimizing the global burden of cervical cancer. In developing countries, women do not approach sufficient screening programs because of the costly procedures to undergo examination regularly, scarce awareness, and lack of access to the medical center. In this manner, the expectation of the individual patient's risk becomes very high. There are many risk factors relevant to malignant cervical formation. This paper proposes an approach named *CervDetect* that uses machine learning algorithms to evaluate the risk elements of malignant cervical formation. *CervDetect* uses Pearson correlation between input variables as well as with the output variable to pre-process the data. *CervDetect* uses the random forest (RF) feature selection technique to select significant features. Finally, *CervDetect* uses a hybrid approach by combining RF and shallow neural networks to detect Cervical Cancer. Results show that *CervDetect* accurately predicts cervical cancer, outperforms the state-of-the-art studies, and achieved an accuracy of 93.6%, mean squared error (MSE) error of 0.07111, false-positive rate (FPR) of 6.4%, and false-negative rate (FNR) of 100%.

## 1. Introduction

Machine learning (ML) and deep learning (DL) are being utilized for brain tumor detection, cervical cancer detection, breast cancer detection, COVID detection, physical activity recognition, thermal sensation detection, and cognitive health assessment of dementia individuals (1–3, 3–10). Advancements in Health Care Industry makes it more effective than traditional diagnosing techniques (11–14). According to medical reports published by Global cancer statistics (15) every year, 493,000 cervical malignancy patients have been added, among which 15% are female malignancy patients. This disease is mainly found in developing countries (16) with an 83% death ratio. Prominent in African countries (17) e.g., Uganda, which ranks fourteenth among the highest incidence of cervical malignancies with 65% of confirmed cases (18).

Human papillomavirus (HPV) contamination is mainly caused by cervical malignancy. HPV contamination is sexually transmitted. Sexual behavior associated with age at first sexual contact and the sexual activity of the accomplice is linked to the increased risk of HPV acquisition. Throughout this case, the cervical malignancy is profoundly preventable through accessible screening and detection than other different sorts of malignancy, and it is critical to actualizing risk expectations. The malignant cervical formation is a malignant tumor. Cervical tissue cells expand and replicate abnormally without regulated cell division and death cause. If the tumor becomes malignant, the cell travels to other areas of the body, such that certain sections become subsequently infected and, in most serious situations, may be avoided through early identification (19). Deaths due to cervical malignancies can be reduced if effective screening strategies are implemented (20). With the fast advancement of present-day clinical innovation and computer technological innovation, various screening and diagnostic strategies depend on computer-aided (CAD) architectures.

A procedure of retrieving applicable information from information sources is known as data mining (21, 22). Real-world information comprises grimy information, for example, inaccurate and incomplete. Along these lines, cleaning and changing crude information to permit a reliable analytic delivery can accurately reflect the outcome (23, 24). It is implemented on the dataset. The cervical cancer dataset acquired for examination contains redundancy, missing values, and noise. Mining methods are considered one of the greatest challenges and significant fields of study in medicine due to the growing importance of health issues (25). The data mining framework can advance the cervical cancer screening process with the help of the knowledge it extracts (26). Across the medical sector, these techniques are helpful not just in finding similarities and correlations between such symptoms but also in predicting diseases. By applying several data mining techniques, continuous research and medical treatment can be immediately recommended, resulting in life-saving, especially for cervical cancer. The first step is pre-processing 80% of information, which plays a vital role as a round of all data mining operations (27).

The random forest (RF) is used to identify essential features in our research that improve the training dataset's quality. RF constructs a forest of tree classifications in which each tree grows on a bootstrap sample data, and the characteristics of every other tree node are selected from a random subset of all characteristics. The final level of the entity is measured by voting across all trees in the forest. Several significant benefits to an RF method makes it the perfect methodology for studying particular biological data in pharmacogenomics research findings. Next, it can accommodate a wide range of both qualitative and quantitative input vectors. Second, it tests the attribute's importance in estimating the type, thereby providing a benchmark for selecting features. Third, RF generates an accurate classifier for unbiased internal generalized analysis during the forest growth process. Finally, RF is relatively stable in the face of etiological variability, and a reasonably low amount of missed data (28).

This article makes the following contributions:

- *CervDetect* implements ML algorithms on the medical data to gain a deeper understanding and to evaluate the risk elements of malignant cervical formation.
- Utilizes Pearson correlation between input variables and the output variable to pre-process the data.
- *CervDetect* uses the RF feature selection technique to select significant features.
- Proposes a Hybrid approach by combining RF and shallow neural network to detect cervical cancer.
- Effectively enhance the detection rate in comparison with state-of-the-art studies.

The rest of the part is arranged as follows. Section 2 presents the literature review. Section 3 presents the dataset and preliminaries. Section 4 presents the proposed methodology. Section 5 examines the result and discussion. Finally, section 6 presents the conclusion and future work.

## 2. Literature Review

In earlier studies, various methodologies based on traditional ML approaches, including k-nearest neighbors (KNN) and K-means clustering and RF, have been utilized for cervical cancer diagnosis (29–31). In 2017, WEN WU1 and HAO developed a clinical decision support network for malignant formation, using a knowledge-based method in conjunction with a rough collection of hypotheses hereditary genetic algorithms calculations in a soft computing model (32). Parallelism, self-learning, and sensitivity to internal failure are artificial neural network (ANN) characteristics. The suggested paradigm takes advantage of these characteristics. Although the information processing capabilities of the rough set theory, and the robust, parallel, and vigorous search are characteristics of the GA's. It still faces high computation problems.

Ashok, Dr. P. Aruna (33) discussed various methods of ML, including support vector machines (SVM), gray level concurrence matrix (GLCM), KNN, convolutional neural networks (CNN), spatial fuzzy cluster algorithms, RF, C5.0, and hierarchical cluster algorithm for feature extraction, segmentation, cell classification, and evaluated in typical parameters, such as dataset volume, disadvantages, and precision. Although it is only best suited for small datasets. Chih-Jen Tseng and Chi-Jie Lu (34) have used image processing, data extraction, and ML techniques to diagnose cervical cancer. The combination of texture and shape features is extracted from each image. Optimal properties are chosen to select reciprocal information, sequential forward selection, and random section selection. Images of cervical cancer are classified using the SVM. Various selection methods are contrasted to determine the proper mechanism suitable for the diagnosis of cervical cancer. The problem faced by SVM is that there is no probabilistic explanation for classification, which leads to very rigid classifications.

In 2006, Reif et al. (35) described RF efficacy in various model genetic and proteomic datasets. RF progress fails to classify related traits based on genetic data and proteomics datasets. Experimental findings indicate that using several data sources is useful where the disease definition is uncertain, and the corresponding data basis for the phenotypic outcome is unknown. This study's findings indicate that RF is exceptional for detecting high-dimensional data vector characteristics with minimal main effects and low heritability, but the problem faced by RF chooses only one attribute at each tree split during construction, strictly epistatic. In 2016, Vidya and Nasira (36) worked on predicting the normal cervix. The cancer cervix is evaluated using practical data mining algorithms. Geetha and Thangamani (37) addressed the imbalanced distribution of data and risk factors for cervical cancer diagnosis.

Anuraga et al. (38) attempted to explore the conditions that affect cancer patients' survival in Makassar, Indonesia. The specimens included in this research contained as many as 38 cancer sufferers. They use the RF to identify tree merger data by combining sample data training. The main problem faced by this technique is that it only achieves 50% of accuracy. The Bandyopadhyay and Nasipuri (39) focused on K-Means clustering to segmentation pre-processed images, and Herlev analysis is carried out. IOU segmentation outcomes, verified ground truth values, and shape attributes are extracted from the segmented nucleus. The identification of the nucleus on the grounds of the shape features is carried out with the RF Classifier's aid, and the contrast is rendered with the other classifiers.

In 2020, Alyafeai and Ghouti (40) build a completely integrated cervical cancer identification and cervical cancer screening pipeline from cervical images. The current pipeline comprises two deep neural network-learning models for automated cervical identification and diagnosis of cervical tumors. The first test detects the cervix area 1,000 times faster than the state-of-the-art data-driven simulations, thus obtaining a detection precision of 0.68 in terms of union intersection (IoU) estimation. Self-extracted characteristics are used in the second model to identify cervical tumors. Such features are trained using two lightweight models focused on co-evolutionary neural networks (CNNs). William et al. (41) performed to reduce the probability of mistake by automating the diagnostic process for cervical cancer from Pap-drug photos. Local adaptive histogram equalization was used for image enhancement.

Brats data (24) is used to localize brain tumors in FLAIR scans of MRI. Bilateral flipping is applied to remove noise, while the Gabor filter bank creates text-ton map images. A superpixel is generated by segmenting out an image superpixel is generated by segmenting-level features include at each superpixel histogram level of texton-map is calculated, and the first-order intensity features are calculated as well. Their major contribution is that they made low-level features significant for the localization of brain tumors at the region level approach by integrating features from texton-map images. The prediction of three classes, namely tumor, non-tumor, and background, was made by giving these extracted features later to the classifier. A cross-validation technique leave-one-out (LOOCV) for the tumor area localization is applied to outperform the existing solution and achieve a dice score of 88%.

In 2021, Wang et al. (42) worked to solve challenges regarding physical security and over-centralized server problems in wireless medical sensor networks (WMSN). Physically unclonable function (PUF) and cutting-edge blockchain technology are combined to propose a reliable and authentic protocol for WSMN. In addition, to deal with biometric information, fuzzy extractor method has been used. For the evaluation of their proposed method, two schemes are used. Their work outperforms the existing studies by achieving higher accuracy through minimizing computation and communication costs. Similarly, Xiong et al. (43) provided a design of blockchain-based ECDSA with fault-tolerant batch verification protocol for blockchain-enabled IoMT. In 2021, Khamparia et al. (1) combined a convolutional network with a variational encoder for data classification. The dimensionality of images data can be reduced by using a variational encoder with a softmax layer with the kernel size of 2x2 and 3x3. Their architecture outperforms the current ML models. Chen et al. (11) developed Cyto Brain that facilitates in subsequent clinical diagnosis, an artificial intelligence (AI) based system. CytoBrain consists of three main modules: (1) to extract only cell images in a whole slide image efficiently, cervical cell segmentation module has been designed. (2) for the cell classification, vgg 16 is used, and a classifier module is designed; Moreover, the last one is the human-aided diagnosis module which can automatically diagnose cervical cancer based on the classification results of cells on a whole slide image.

## 3. Dataset and Preliminaries

The dataset^1^ used has been released in The University of California's database at Irvine (UCI). The collection contained existing patient history, practices, and procedures and demographic statistics for 858 instances with 32 features per scenario. The dataset contains several missed features since others are incomplete instances, which were chosen not to tackle any privacy concerns, as privacy and security are the common issues in healthcare record frameworks (44–48). Table 1 displays the dataset characteristics and the missed value for each function.

**Table 1:** Dataset description.

**Serial number**	**Features names in dataset**	**No of missing values**
1	“Number of sexual partners”	26
2	“First sexual intercourse”	7
3	“Num of pregnancies”	56
4	“Smokes”	13
5	“Smokes (years)”	13
6	“Smokes (packs/year)”	13
7	“Hormonal Contraceptives”	108
10	“Hormonal Contraceptives (years)”	108
11	“IUD”	117
12	“IUD (years)”	117
13	“STDs”	105
14	“STDs (number)”	105
15	“STDs:condylomatosis”	105
16	“STDs:cervical condylomatosis”	105
17	“STDs:vaginal condylomatosis”	105
18	“STDs:syphilis”	105
20	“STDs:pelvic inflammatory disease”	105
21	“STDs:genital herpes”	105
22	“STDs:molluscum contagiosum”	105
23	“STDs:AIDS”	105
24	“STDs:HIV”	105
25	“STDs:Hepatitis B”	105
26	“STDs:HPV”	105
27	“STDs: Time since first diagnosis”	787
28	“STDs: Time since last diagnosis”	787
29	“Age”	0
30	“STDs: Number of diagnosis”	0
31	“Dx:Cancer”	0
32	“Dx:CIN”	0
33	“Dx:HPV”	0
34	“Dx”	0
35	“Hinselmann”	0
36	“Schiller”	0

## 4. Proposed Methods

**Figure 1:**
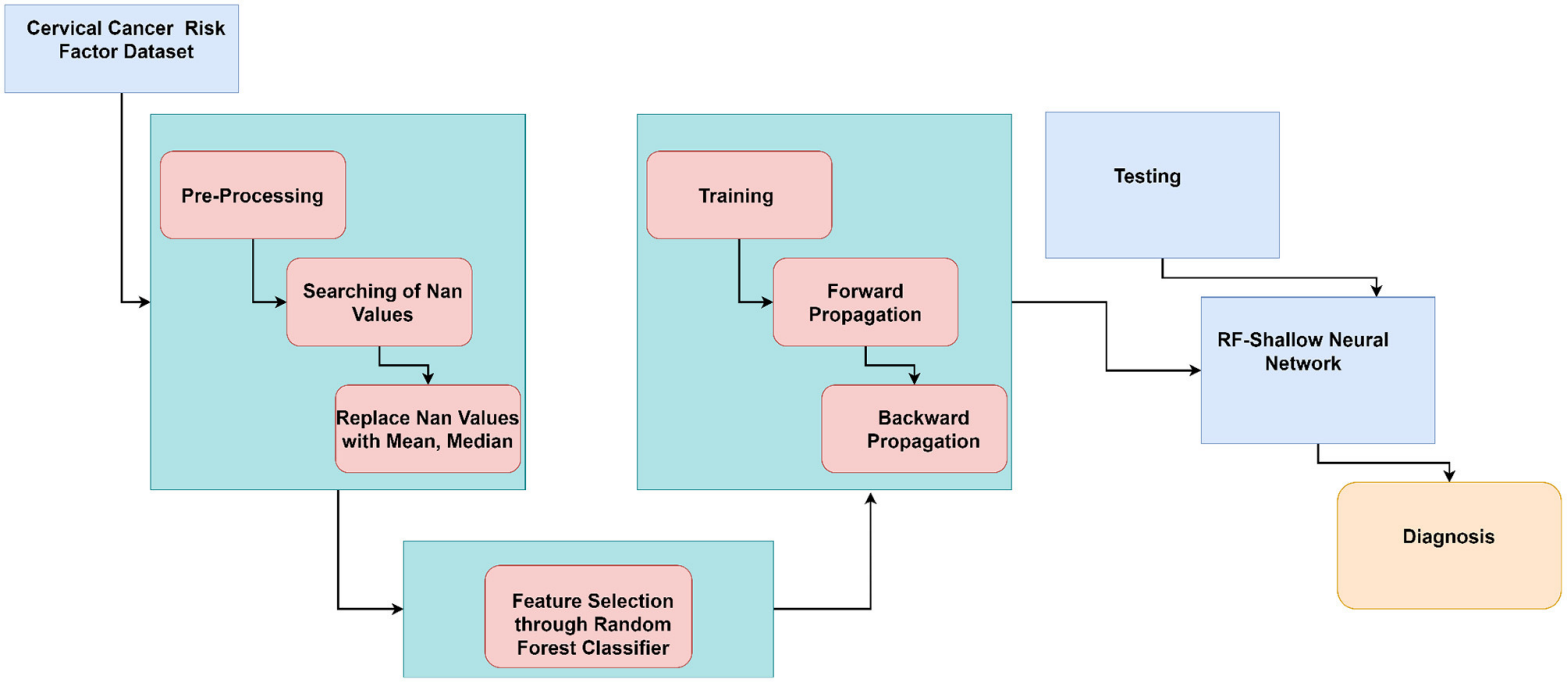
Block diagram of proposed work flow.

Figure 1 illustrates the flow of our work in a block diagram consisting of pre-processing and classification of cervical cancer. The proposed approach consists of pre-processing and classification of cancer using ML algorithms and deep learning algorithms.

Algorithm 1 shows the working of *CervDetect*. Random samples have been collected from the input cervical cancer risk factor dataset. For each collected sample, a decision tree has been made to make a prediction. Counting has been done for each final score, and the features are ranked according to their importance. Selected features are input to the shallow neural network where *x*_*i*_(i=1, 2, …  n) are the number of input features, *w*_21_ and *w*_22_ are the weights and B1 is constant. y stores the features convolved with a randomly initialized filter matrix. V store results of applying a linear transformation on y. *O*_1_ stores the output generates after applying a sigmoid function on y, and e calculates the change in error concerning weights. Infeed backward change error to the weights has been calculated with the help of the chain rule, and the weights are updated.

**Algorithm 1:** *CervDetect* Algorithm.

**Input**: Cervical cancer risk factor dataset.
1: Begin with the collection of random samples from a dataset.
2: First, this algorithm must generate a decision tree for each sample.
3: The prediction will come from the decision tree.
4: Counting will be carried out in this stage for each final score.
5: Ultimately, pick the most elected outcome of the prediction as to the outcome of the prediction.
6: **Output**: Rank features with according to their importance.
7: **Input**: selected features input to shallow neural network *x*_*i*_(i=1, 2, 3, 4…n)
8: Initialization is the first step after the configuration of the neural network
9: Initiate all weights *w*_21_, *w*_22_ with a random number from the usual distribution, i.e. N (0, 1).
10: Put all of the bias nodes B1 = B2 = 1.0.
11: Feedforward
12: **for** *I* ∈ *l* **do**
13: each of x input have weights w21, w22 …
14: y=*w*_21_ *x*_1_, *w*_2_2 *x*_2_, …,*w*_*m*_ *x*_*i*_,
15: v= sigmoid(y)
16: *o*_1_= *y*_*v*1_ *w*_21_
17: *o*_*v*_ = *o*_1_
18: e=$\frac{1}{n}\overset{2}{\underset{i=1}{\sum }}$ (*y*_*i*_-ovi)^2^
19: **end for**
20: Feed backward
21: **for** *i* ∈ *k* < *l* **do**
22: Partial derivative of the e to the weight adjusted $w_{i}^{k+1}$ *w*_*ik*_-Δ $/\left(\varphi w_{k^{i}}\right)$
23: weight update *e*_*i*_=*e*_*i*_ Δ+$w_{i}^{k+1}$
24: **end for**

### 4.1. Data Pre-processing

The dataset regarding cervical cancer has many missed values. A missing value can imply a variety of differences. Records with missed values could be included, omitted, or the mean of the variable can be replaced for missing numerical characteristics or with the most frequent value in the case of categorical features. The technique of eliminating the amount of data with missing rows of values decreased from 858 to 737 when applied. We aim to scale the number of features but not the number of input vectors used in the dataset. The following steps are included to pre-process data and make it suitable input for the classifier.

**Figure 2:**
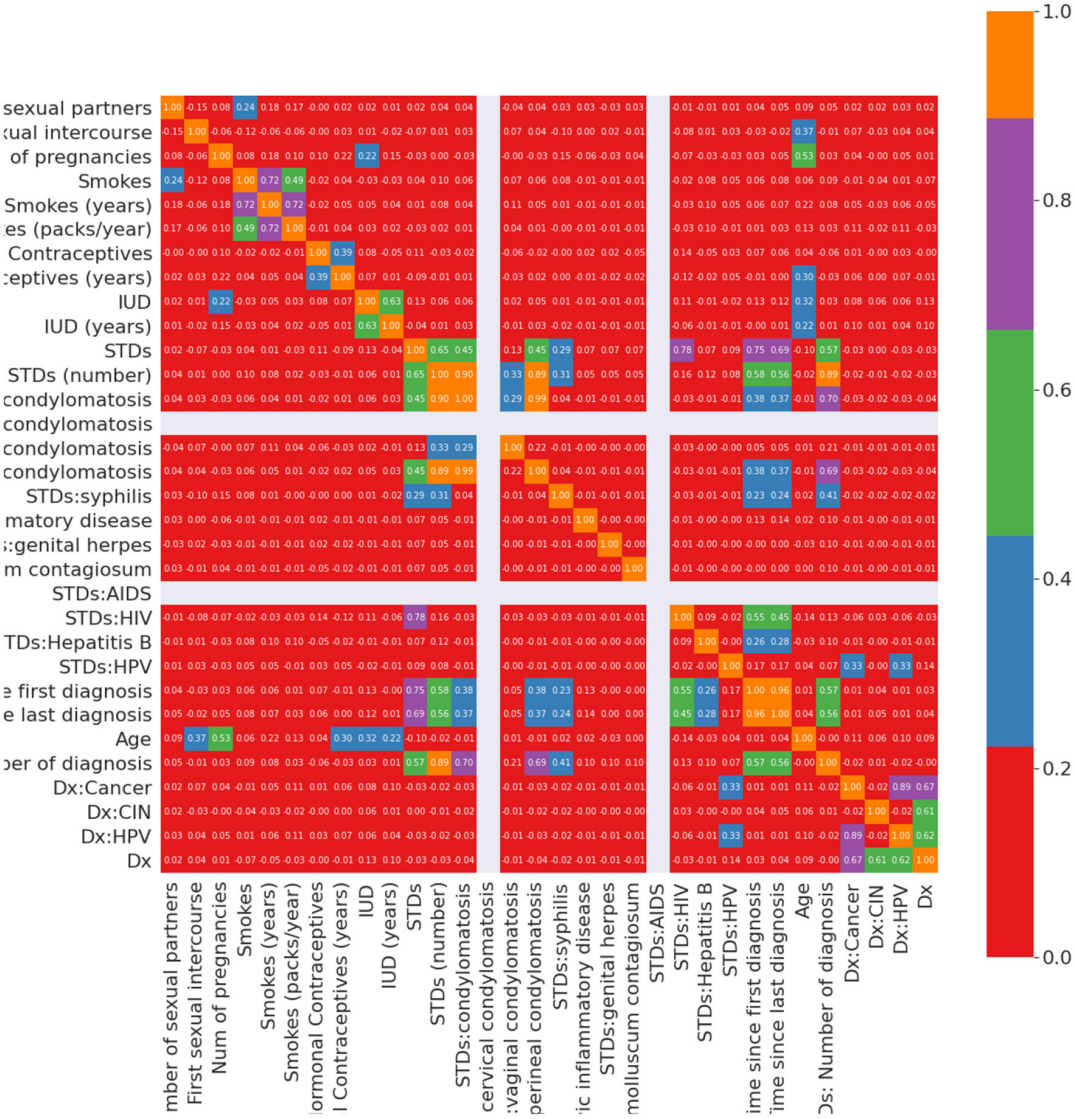
Correlation between input variables after handling missing values.

1. Nan: To see relations between variables to treat Nan. Nevertheless, according to the data, the scale of more than 100 Nan values may impact results. We fill features with a median value of less than 100 Nan values in them with median values.
2. Hormonal contraception: There are so many Nan values during the data diagnosis information. Because of this, we cannot calculate the impact of this information, and must eliminate them. Instead, using the Pearson correlation, we will decide which attribute influences hormonal contraceptives. We fill Nan values with correlated attributes according to the resultant heat map. If the patient is older than the mean sample or the amount of pregnancy is less than the mean, the patient may take hormonal contraceptives. Nan values in HC (years) fill with median values using HC attributes.
3. IUD: Using the Pearson correlation, we can decide which attribute is the “IUD” influencer. Age and amount of maternity factors have an impact on the IUD feature. This indicates that 80% of patients taking IUD are older than the average age. 70% of patients who do not take IUD have fewer births than the mean amount of pregnancies. We can fill in the remaining Nan IUD feature values. With the IUD (years) rule, we can fill the -1 values with the IUD rule. If the patient takes IUD, then the UID (years) will be non-zero, so we need to adjust it to mean values.
4. STDs: condylomatosis and STDs: vulvo-perineal condylomatosis has the “STD” effect. We never consider “STD (number)” and “STDs: number of diagnoses” as they are the same attributes as “STDs.” Based on our analysis of STDs, we can conveniently fill the Nan values of 1 or zero, and if the individual has either of the STDs, then, the STDs should be 1, the other one will be 0.
5. STD diseases: According to information, 73% of non-smoking patients have STD. We do not have Nan values 75% of patients who do not take IUD are even STDs. Even STDs (number) are the same attribute as STDs. The median case is not helpful because we use mean values for replacement. As per the heat map and our domain awareness, all STD diseases rely on the STDs function and STD (number). We fill Nan values using median for specific values since all STDs rely on certain STDs, and we cannot be assured of a person's disease.
6. AIDS: The correlation function does not give us any hints. However, we recognize that AIDS is also a condition of STDs. So, we fill in the Nan values.
7. STDs-Hepatitis B: This aspect is the result of the STDs-HIV factor. Here we have one patient with a disease, and this importance is negligible compared to the population. The value of Nan is filled with 0.
8. STDs-HPV: The positive values of HPV are not enough. Zero values cannot be determined by using significant attributes, and we filled the value of Nan with 0.
9. STDs-time after the first diagnosis and STDs-time after the last diagnosis: When the patient's STDs are negative, the first and last diagnoses cannot be positive. This assumption fills the Nan values in these attributes. Figure 2 illustrates the Pearson correlation between attributes in the cervical cancer risk factor dataset after handling missing values.

### 4.2. Random Forest

The RF model utilizes a sequence of decision trees, where each tree in the forest has been trained using a bootstrap sample of data items, so each tree splitting attribute is picked from a random subset of features. The categorization of patterns shall be based on the collective vote of all trees in the forest. Attribute importance is measured as the decreases in the node's impurity, weighted by the likelihood of approaching the node. The likelihood of the node can be determined by the number of instances that enter the node, separated by the total number of instances. The greater the value, the more important the characteristic is. In Scikit-learn Decision tree, approximates the value of the nodes using Gini significance, considering just two child nodes (binary tree):


(1) $$nodei_{j=}Weight_{j}C_{j}-W_{l\left(j\right)}C_{l(j)}-W_{r\left(j\right)}C_{r(j)}$$

In Equation (1), *nodei*_*j*_= Importance of j node; *Weight*_*j*_= Weighted samples that reach at node j; *C*_*j*_ = Impurity of jth node; l(j)= From left split child node on jth node; r(j)= From right split child node on jth node. Each feature importance calculated at decision tree is given by Equation (2):


(2) $$featureimportancei_{i}=\frac{\sum j\;n_{i(j)}}{\sum kn_{ik}}$$

Where, ∑j= splitting of a feature I at node j; *n*_*i,j*_= node j importance; ∑k= all nodes. In Equation (3), normalizing these values between 0 and 1 is done by taking the sum of all feature's importance values and then dividing them.


(3) $$normalizedfi_{i}=\frac{fi_{i(feature\;importance)}}{\sum j\;fi_{j}(all\;features\;)}$$

In Equation (4) at the RF stage, the final feature of importance is the sum, including all trees. Estimating an attribute's significance at each tree is calculated and divided by the overall tree species.


(4) $$Random\;forestfi_{i}\;\frac{\sum j\;normalizedfi_{ij}}{T}$$

**Figure 3:**
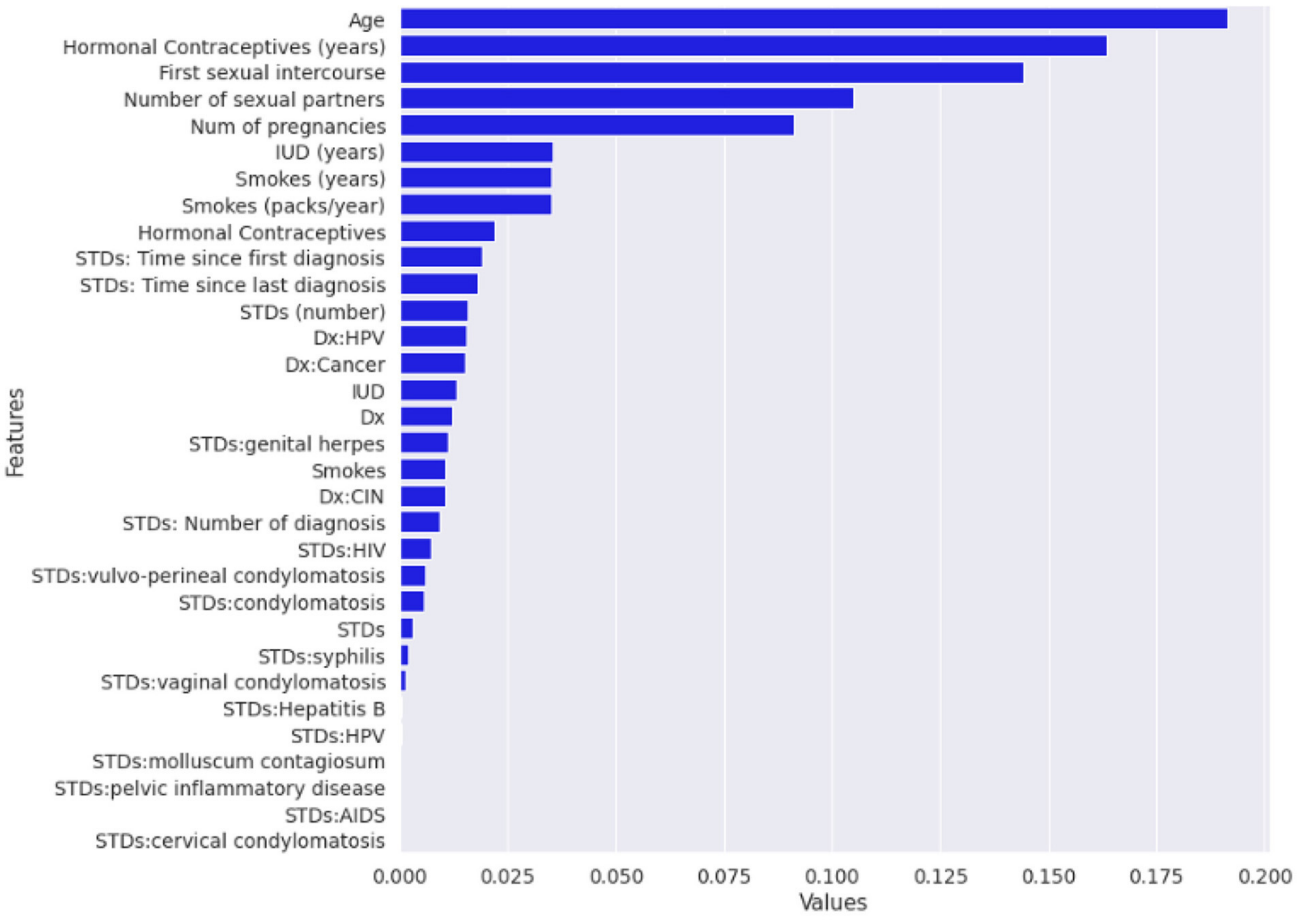
Graph plotted after applying random forest (RF) algorithm for feature importance.

Normalized *fi*_*i,j*_ is the normalized feature importance for I feature at jth tree, and T is the total number of trees in the forest. Figure 3 illustrates the feature importance graph that improves the quality of the training dataset.

### 4.3. Artificial Neural Networks

Artificial neural network models are influenced by the human brain structure, which interconnects many biological neurons that are important for maintaining coherent communication. The ANN architecture is computer-based and consists of a variety of simple parallel processing units (49). It is a common statistical technique that can analyze exact relationships between variables. There are several parallel layers in the ANN models, and each layer consists of several neurons. Input, hidden, and output layers are three different types of layers. No calculations are conducted on the input layer. Only feature variables are input. The hidden layer is the key part of the ANN and includes different neurons commonly detected by checking and error. The linear and non-linear functions for the hidden layer neurons are required. The first stage is where the hidden layer neurons receive the input variables compounded by the corresponding correlations (weights), and the second cycle is followed by a non-linear induction system, usually a sigmoid. A fully connected ANN consists of neurons. The neurons are split into layers composed of one input layer, one output layer, and numerous hidden layers with the contribution of each layer in the next layer as an input in our research, and we concentrate on neural networks with only one layer of hidden data, hidden neurons, and a single output.

### 4.4. Shallow Neural Network Architecture

**Figure 4:**
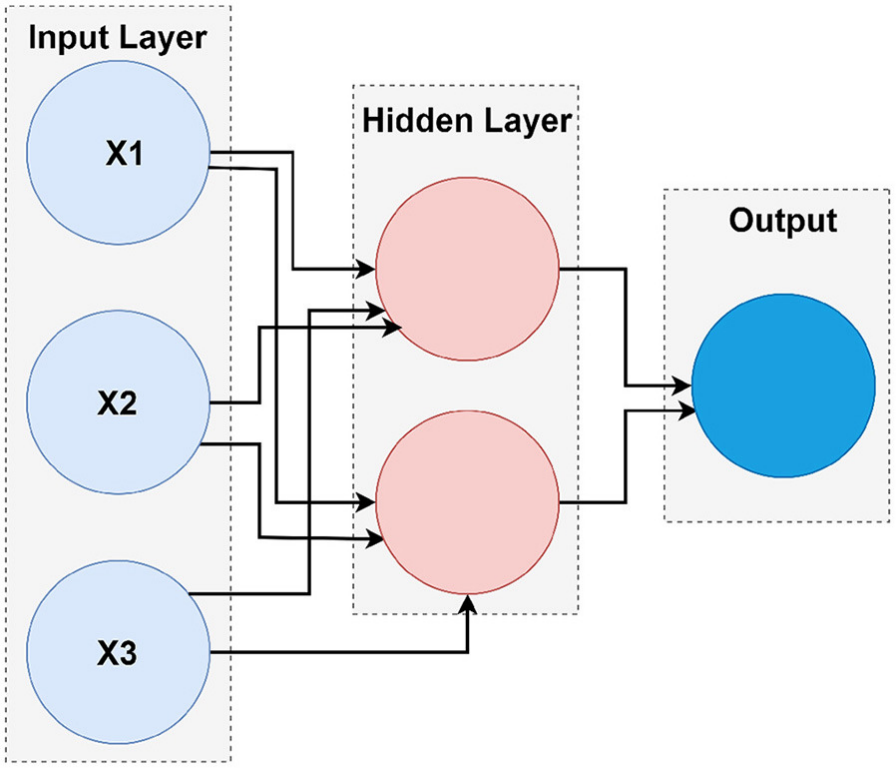
The architecture of shallow neural network.

Different types of neural networks, both shallow and deep, have been built. The terms “shallow” and “deep” refer to the neural network with a minimum number of layers typically known to have a common hidden layer. Deep neural nets are used by neural networks that contain several deep layers. These types of networks execute various tasks, and the fundamental framework of shallow networks enables them to do so. Almost all shallow networks had an input layer, a single hidden layer, and an output layer. The number of hidden nodes in the layer is the only different hyper-parameter. Networks use online learning instead of batch learning, which uses simple backpropagation and gradient descent. Besides, a scaled conjugate gradient descent (SCG) backpropagation algorithm was used in shallow networks. Figure 4 is the fundamental model for shallow neural networks.

The neurons are the atomic component of the neural network. For input data, the output is defined as well as the output is passed to the next layer as an input vector. A neuron may be thought of as a fusion of different segments:

1. The first section calculates the output Y, using input parameters and weights.
2. The second section executes the activation on Y to give the final performance of neuron A.

The hidden layer consists of several neurons, which each executes below mentioned 5 and 6 equations. Those two neurons found in the hidden layer of the external neural network are evaluated as follows:


(5) $$Y_{1}^{\left[1\right]}=w_{1}^{\left[1\right]}Tz+\;b_{1}^{\left[1\right]},a_{1}^{\left[1\right]}=\;\sigma \left(y_{1}^{\left[1\right]}\right)$$

(6) $$Y_{2}^{\left[1\right]}=w_{2}^{\left[1\right]}Tz+\;b_{2}^{\left[1\right]},a_{2}^{\left[1\right]}=\;\sigma \left(y_{2}^{\left[1\right]}\right)$$

Where the superscript number [i] is the number of the layer, and the subscription number j is the number of the neurons in a particular layer. Y is an input vector made up of three components.

*W*_*i*_ represents the weights connected to each input variable, and bi represents the bias factor connected to each line, hidden line, and output layer. Z[i]j is just the intermediate output associated with the j neurons found in the ith layer. A[i]j is the final output associated with the neuron j in the ith layer.

Sigma is a function of activation that squashes the input value into the range of (0,1). Mathematically, this is described as in Equation (7):


(7) $$\sigma =\frac{1}{1+\;e^{-yi}}$$

Equation (8) represents all Z intermediate outputs in a single multiplication matrix.


(8) $$Z^{1}=X^{[1]T}X+b^{1}$$

(9) $$A_{1}^{\left[1\right]}=\;\sigma (Z^{1})$$

The above Equation (9) represents all activation A in a single multiplication matrix.

To calculate the output for an input vector y, the following steps are performed as mentioned in below (10), (11), (12), and (13) equations. These steps can also be called feed-forward propagation.


(10) $$Z^{1}=W^{[1]T}Y+b^{1}$$

(11) $$A_{\text{1}}^{\left[\text{1}\right]}=\;\sigma (Z^{\text{1}})$$

(12) $$Z^{2}=W^{[\text{2}]T}A^{1}\text{+}b^{\text{2}}$$

(13) $$Z^{final}=A^{\left[2\right]}=\;\sigma (Z^{2})$$

Where the Equation (10) measures the Z (18) intermediate output of the first hidden layer. The Equation (11) is used to measure the final production A (18) of the first hidden layer. The Equation (12) calculates the Z intermediate value of a processing layer Z. The Equation (13) calculates the end product A of the output layer, which is now the result of the whole neural network. When the results are obtained from each neuron's hidden layer, they are transferred to the next layer, where each neuron in the output layer finalizes the values.


(14) $$Y_{1}^{\left[2\right]}=w_{1}^{\left[2\right]}out\left(y_{1}\right)+w_{2}^{\left[2\right]}out\left(y_{2}\right)+..+w_{14}^{\left[2\right]}out(y_{14})+\;b_{2}^{\left[2\right]}$$

(15) $$a_{1}^{\left[2\right]}=\;\sigma \left(Y_{1}^{\left[2\right]}\right)\;$$

(16) $$Y_{15}^{\left[2\right]}=w_{30}^{\left[2\right]}out\left(y_{1}\right)+w_{\text{31}}^{\left[\text{2}\right]}out\left(y_{2}\right)+..+w_{43}^{\left[2\right]}out(y_{15})+\;b_{2}^{\left[2\right]}$$

(17) $$a_{50}^{\left[2\right]}=\;\sigma \left(Y_{50}^{\left[2\right]}\right)$$

Equations (14– 17) are used to determined the error after the results are obtained for each neuron in the output layer. When this is the critical error, it will avoid back-propagating to change the previous weights to get the minimal error in the feed-forward process.

## 5. Results and Discussion

In our work, the most significant performance measure for cervical cancer diagnosis accuracy is used to calculate the RF-shallow neural network's performance. The true positive (TP) is identical to those rejected, representing the number of cancer patients marked as Biopsy. False-positive (FP) is the inverse to deny wrongly and represents normal patients as cancer patients. The true negative (TN) is equal to those correctly identified, representing the number of normal patients identified as normal. False-negative (FN) is equal to any incorrectly identified, representing the number of cancer patients identified as normal patients. Table 2 shows the confusion matrix.

**Table 2:** Confusion matrix of *CervDetect*.

**Predicted class /Actual class**	**Biopsy**	**Normal**
Biopsy	TP	FN
Normal	FP	TN

Accuracy: the proportion of the number of patient records correctly categorized against the overall number of patient records in the dataset as shown in Equation (18).


(18) $$AC=\frac{TP+TN}{Tp+TN+FP+FN}$$

True positive ratio (TPR): it is identical to detection rate (DR). TPR indicates the proportion of the number of patient records correctly identified over the overall patient records, as shown in Equation (19).


(19) $$TPR=\frac{TP}{TP+FN}$$

False positive ratio (FPR): the ratio of the numbers of incorrectly declined records divided by the cumulative amount of total record as shown in Equation (20)


(20) $$FPR=\frac{FP}{FP+TN}$$

In this study, we have proposed a new method called RF-shallow neural network to diagnose cervical cancer as the effect of the wrong diagnosis in cervical cancer or vice versa is high. Data mining provides tools and techniques to derive important data from a huge dataset by analysis. Throughout this study, ML models (RF and shallow neural network) were used for cervical cancer diagnostics to demonstrate the importance of building the model with data cleaning, the replacement of null values, and the implementation of a feature selection procedure to achieve a better accuracy prediction for an optimal function subset. This methodology used ML methodologies for cervical cancer data to assess the efficacy of classifier models by considering all medical records. Dataset missing records are filled by replacing missing values in rows with their mean and median by finding the Pearson correlation between variables. The RF's implementation as a feature selection technique and the shallow neural network training by an optimum subset of features was chosen based on the variables' value. In addition to the one target attribute biopsy for cervical cancer diagnosis, the following attributes in Table 3 have been defined as more important features among the cervical cancer risk factors dataset.

**Table 3:** Selected optimal features.

**Serial no**	**Important features according to their rank**
1	“Age”
2	“Hormonal Contraceptives (years)”
3	“First sexual intercourse”
4	“Num of pregnancies”
5	“IUD (years)”
6	“Smokes (years)”
7	“Smokes (packs/year)”
8	“Hormonal Contraceptives”
9	“STDs: Time since first diagnosis”
10	“STDs: Time since last diagnosis”

**Figure 5:**
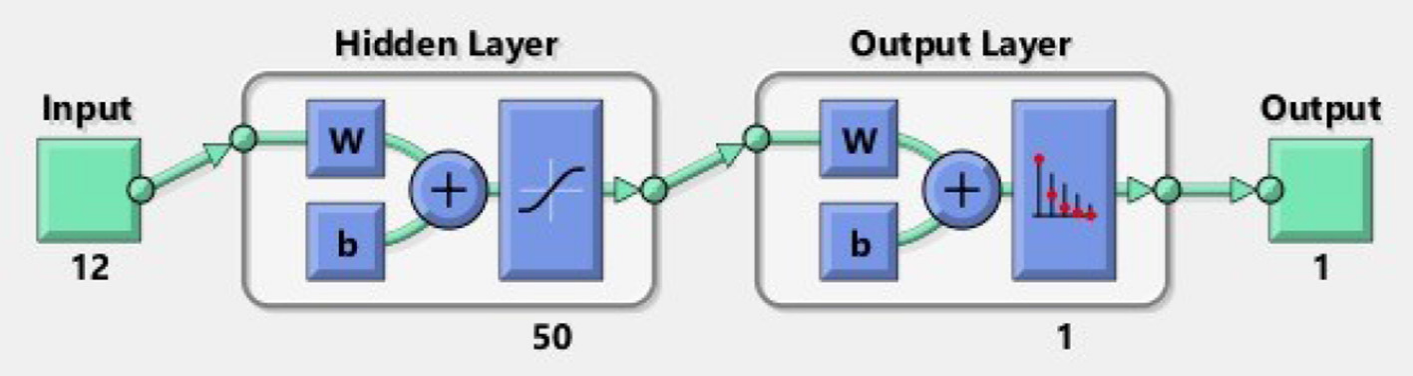
Shallow neural network.

A shallow neural network consists of one input layer, one layer of hidden neurons, and a single output layer, as shown in Figure 5. Neural pattern recognition uses the scaled conjugate gradient algorithm. The value of one class, "0," is treated as a negative test for a cervical cancer patient, and the value of another class, "1," is considered a positive measure for a cervical cancer patient and recommended for biopsy.

A total of 12 optimum features input space has been created after their importance and ranking. We took 70% of the data from 859 records for training, 15% for validation, and 15% for testing. To obtain the results, we used 50 hidden neurons and 1 epoch. Table 4 shows the findings we obtained from ANN using scaled conjugate gradient using minimized cross-entropy, correct true positive rate, false-positive rate, and accuracy.

**Table 4:** Accuracy table.

**serial no**	**Performance measures**	**Performance%**
1	Accuracy	93.6%
2	TPR	100%
3	FPR	100%

**Figure 6:**
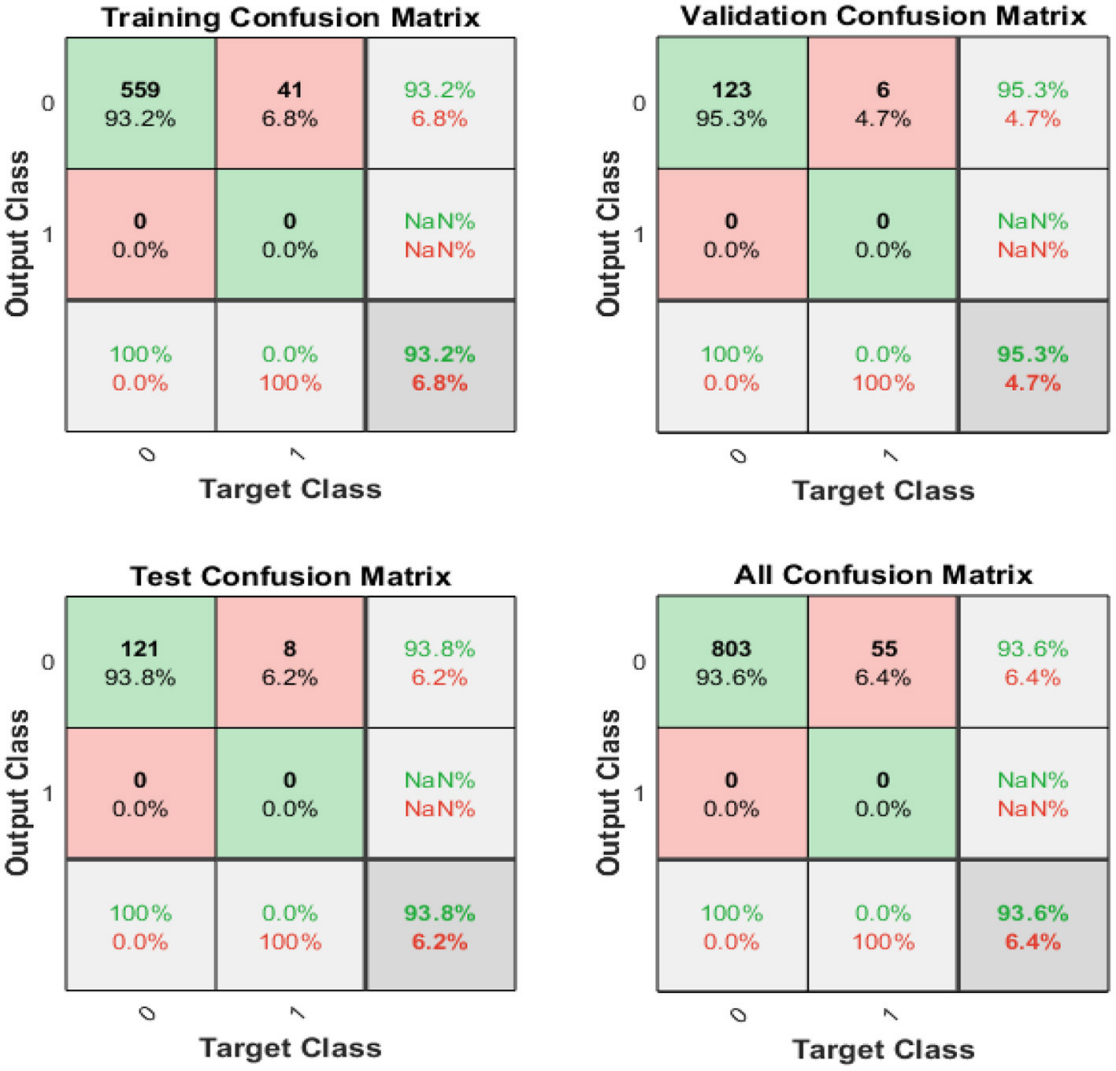
Confusion matrix of training, testing, and validation.

Figure 6 displays the confusion matrixes for validation, testing, and training, and the three pieces of information merged. The network findings are promising because we can conclude with many correct responses in green squares and a minimal number of wrong responses in red squares. The lower squares indicate the actual precision.

**Figure 7:**
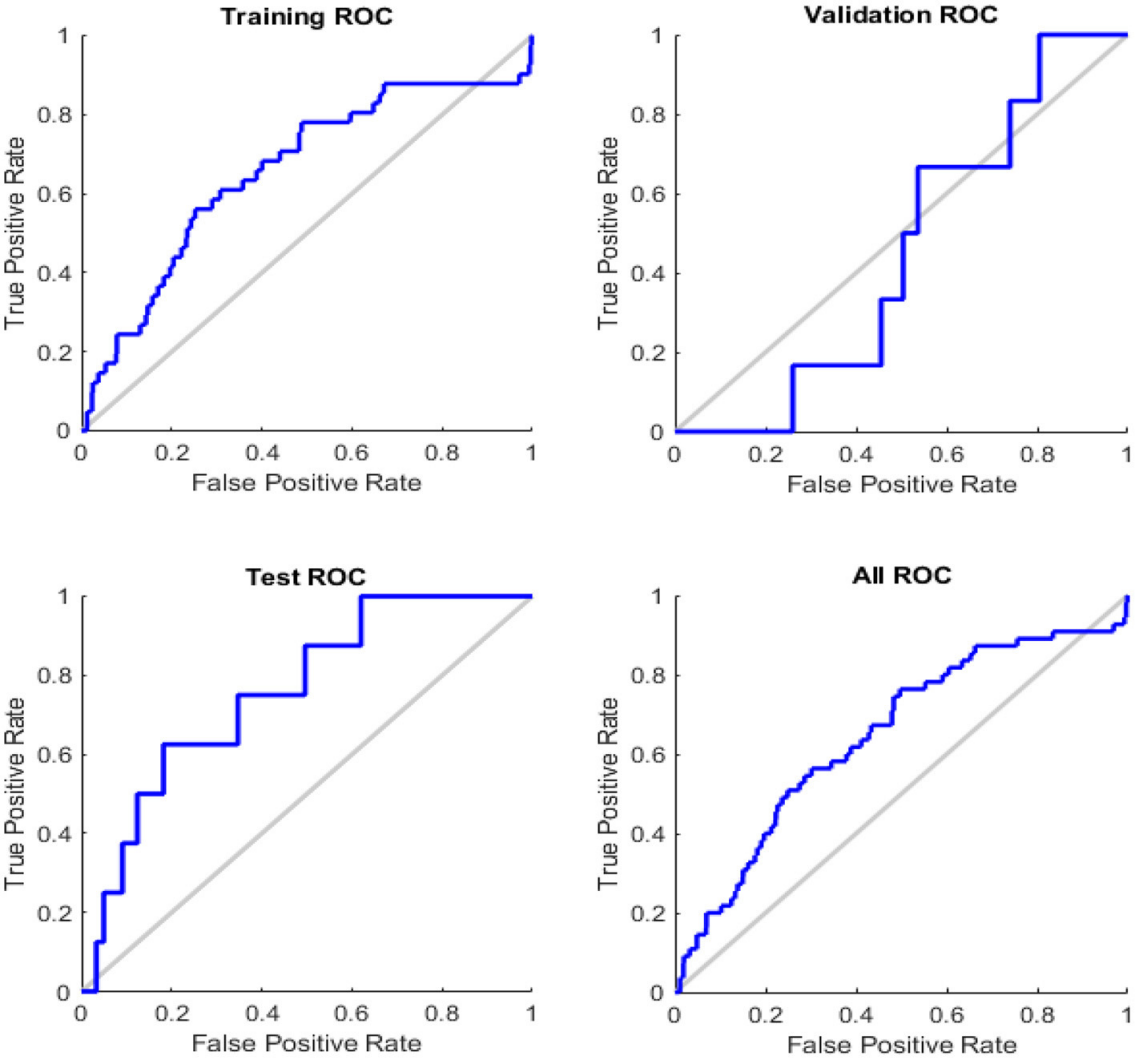
Roc curve of testing, training, and validation.

Figure 7 illustrates the receiver operating feature (ROC) curve. The colored lines in each axis represent the curves of a ROC. The ROC curve is a graphical representation of a true positive (sensitivity) vs. the false positive (1-specificity) in which the threshold has been changed. The optimal search has shown points in the upper left corner with 93% accuracy.

**Figure 8:**
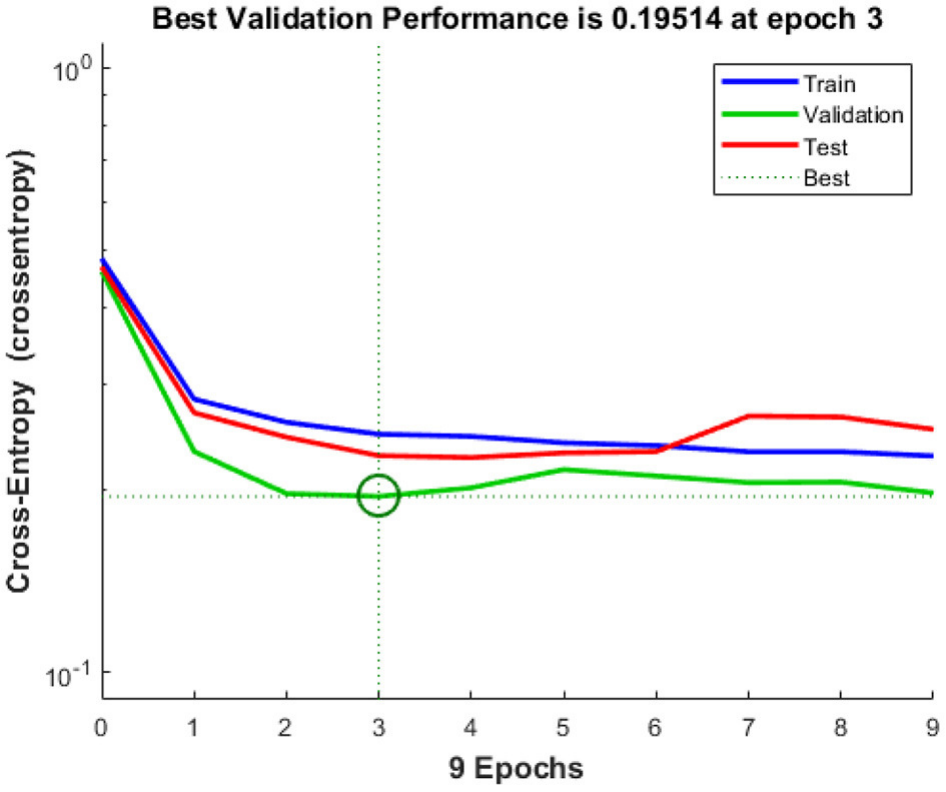
Cross entropy.

There have been several lines, train, validation, testing, and best in this study, as shown in Figure 8. In general, the ideal (dotted) line is that the other will lie on or above this (dotted) line, and we can conclude that the training has been carried out effectively. If all of the 3 (training, validation, and testing) lines cross or travel past the ideal (dotted) path, this implies that convergence has been completed. If this is not the case, retrain the network.

**Figure 9:**
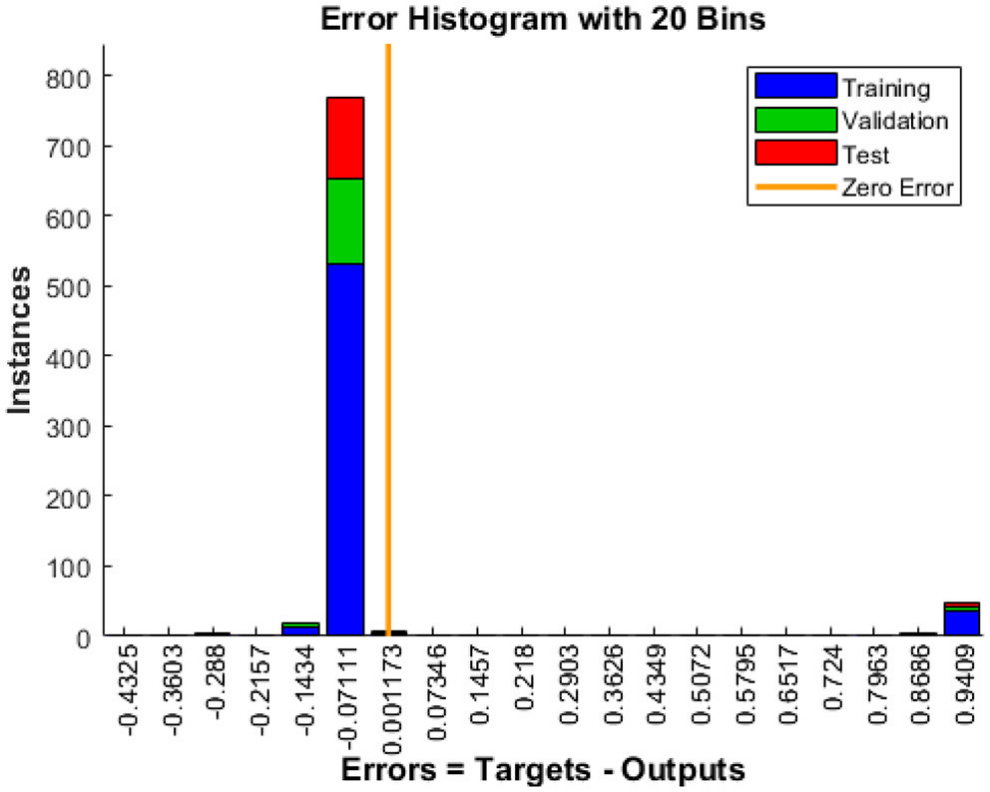
Error histogram.

When the model is tested for more than 129 tests, the histogram displayed in Figure 9 shows the error that occurs during the test process. The distribution of the error size of the histogram shows how well the neural network matches the results. It indicates errors that the solution to mean squared error (MSE) in our scenario is good. More than 500 events are utilized for training purposes. Approximately, 129 are used for validation, and the remaining 129 is used to check which indicates an error of 0.07111.

**Figure 10:**
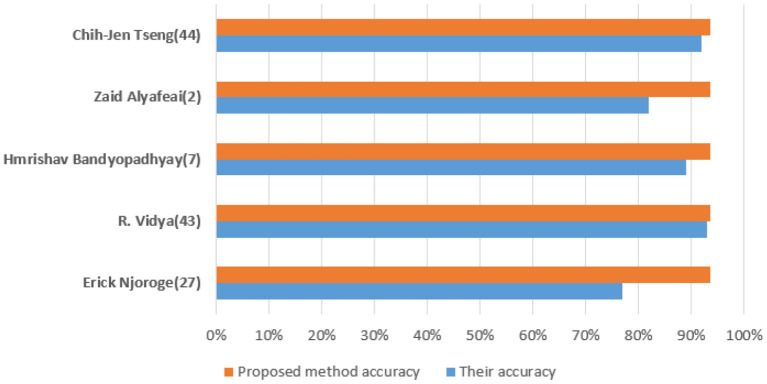
Comparative analysis with existing works.

**Figure 11:**
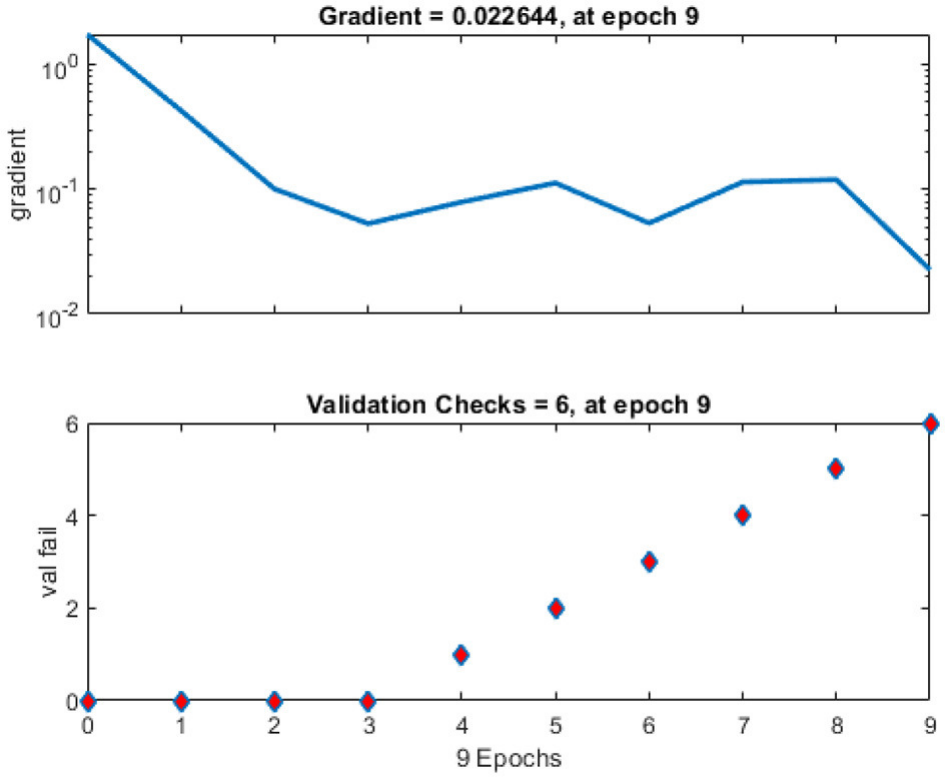
Training state of neural network for cervical cancer diagnosis.

Figure 10 shows a training state that represents the actual progress/state of the training at a given period when the training is in progress. In our situation, 6 validation errors are stated, i.e., unless 6 validation tests are performed, then training will end. Validation test error implies that the dataset has specific issues; a training algorithm cannot recognize certain instances. This indicates that due to dataset issues, validation test errors may be produced. In our case, 6 validity tests are included. As shown in Figure 11, modified *CervDetect* achieved 93.6% that outperforms the existing work.

## 6. Conclusion and Future Work

This study provides a risk factor for cervical cancer. The cure percentage, the number of female cases recovered from cancer may be improved by recognizing risk factors for cervical cancer. Data mining techniques in the medical industry have an immense capacity to develop diagnostic and prognosis indicator applications that can aid in the proper initial treatment of life-threatening diseases. With various data mining techniques, it is important to enhance the standard of care in hospitals and maximize patients' recovery rates. The outcomes of the data reduction also continued to increase the efficiency of the methods. Generally, the less critical features used in the classification process, the stronger the external neural network's efficiency. In this article, we present *CervDetect*, a hybrid approach that combines RF and shallow neural network to understand the risk elements of malignant cervical formation by deploying machine learning algorithms on medical data. *CervDetect* utilizes Pearson correlation between input variables and the output variable to pre-process the data. The RF feature selection technique is used to select significant features. *CervDetect* effectively enhances the detection rate in comparison with state-of-the-art studies. Results show that the proposed approach achieved an accuracy of 93.6%, MSE error of 0.07111, FPR of 6.4%, and FNR of 100%. For future work, applying a sequential model to this dataset, such as a recursive deep neural network, can be useful for diagnosis. Due to the severity of the disease, cervical cancer is chosen as a starting point. The same work delineated here could be extended to other gynecological cancer predictions and other disease entities. Thus, the provided approach *CervDetect* showed optimal performance accuracy with the optimum features dataset.

## Data Availability Statement

The original contributions presented in the study are included in the article/supplementary material, further inquiries can be directed to the corresponding author/s.

## Author Contributions

SA and MM: conceptualization. MM: data curation. SA: formal analysis, investigation, methodology, and software. MR: funding acquisition. MG and MR: project administration, resources, and writing—review and editing. MG and SA: supervision. MM and MG: validation and visualization. All authors contributed to the article and approved the submitted version.

## Conflict of Interest

The authors declare that the research was conducted in the absence of any commercial or financial relationships that could be construed as a potential conflict of interest.

## Publisher's Note

All claims expressed in this article are solely those of the authors and do not necessarily represent those of their affiliated organizations, or those of the publisher, the editors and the reviewers. Any product that may be evaluated in this article, or claim that may be made by its manufacturer, is not guaranteed or endorsed by the publisher.

## Footnotes

^1^ https://archive.ics.uci.edu/ml/index.php
